# Feasibility, Efficacy, and Safety of Percutaneous Muscle Biopsies in Patients With Chronic Liver Disease

**DOI:** 10.3389/fphys.2021.817152

**Published:** 2022-02-15

**Authors:** Jonathan I. Quinlan, Amritpal Dhaliwal, Felicity Williams, Sophie L. Allen, Leigh Breen, Carolyn A. Greig, Janet M. Lord, Matthew J. Armstrong, Ahmed M. Elsharkawy

**Affiliations:** ^1^NIHR Birmingham Biomedical Research Centre, University Hospitals Birmingham NHS Foundation Trust, University of Birmingham, Birmingham, United Kingdom; ^2^School of Sport, Exercise and Rehabilitation Sciences, University of Birmingham, Birmingham, United Kingdom; ^3^Institute of Inflammation and Ageing, University of Birmingham, Birmingham, United Kingdom; ^4^MRC-Versus Arthritis Centre for Musculoskeletal Ageing Research, University of Birmingham, Birmingham, United Kingdom; ^5^Liver Unit, Queen Elizabeth Hospital Birmingham, Birmingham, United Kingdom

**Keywords:** chronic liver disease (CLD), muscle biopsy, non-alcoholic fatty liver (NAFLD), end stage liver disease, liver disease

## Abstract

**Introduction:**

Sarcopenia is present in many chronic disease states including decompensated end stage liver disease (ESLD) and non-cirrhotic non-alcoholic fatty liver disease (NAFLD). Sarcopenia in ESLD can negatively impact quality of life and increase mortality. Despite this, very little is understood about the mechanisms of sarcopenia in these conditions. One key reason for this is the reluctance to undertake percutaneous muscle biopsies due to the perceived increased risks. ESLD can induce thrombocytopaenia and coagulopathy which significantly increases the risk of bleeding. In addition, patients with either NAFLD or ESLD often have co-morbidities that would require additional care and risk assessment. Thus, the aim of this study was to establish an effective and safe protocol for the implementation of percutaneous muscle biopsies in patients with NAFLD and ESLD.

**Methods:**

A total of 47 patients with ESLD and 9 patients with non-cirrhotic NAFLD were recruited from the Liver Unit, Queen Elizabeth Hospital (Birmingham, United Kingdom). A total of 71 percutaneous vastus lateralis biopsies were attempted over two study visits. A vigorous safety screening occurred prior to and during each visit and a strict protocol was followed to mitigate against complications and risk.

**Results:**

A total of 85% of patients consented to the muscle biopsy at either visit (48/56). A total of 9% of consented biopsies could not occur due to medical considerations, including high international normalised ratio (INR) (*n* = 3) and the use of aspirin (*n* = 4). Muscle tissue was obtained from 90% of attempts, with a mean average yield (wet weight tissue) of 98.1 ± 52.9 mg.

**Conclusion:**

Percutaneous muscle biopsies are both feasible and yield sufficient tissue in an ESLD population. The procedure is effective for obtaining muscle tissue whilst also safe, with only one adverse event. This study provides evidence for the successful use of muscle biopsies in this population, even in consideration of disease specific complications, medications, and comorbidities.

## Introduction

The loss of muscle mass, function and quality is a condition known as sarcopenia (Cruz-Jentoft et al., 2019). Sarcopenia has typically been considered an age-related condition; however, it is also present in many chronic disease states. As such, sarcopenia can be subdivided into two categories: primary and secondary sarcopenia (Cruz-Jentoft et al., 2019). Primary sarcopenia is the result of the natural ageing process, i.e., largely occurs due to ageing alone. In contrast, secondary sarcopenia occurs due to a pre-existing condition or disease which consequently negatively impacts skeletal muscle. Secondary sarcopenia is often present in chronic disease states such as chronic liver disease (CLD); specifically non-alcoholic fatty liver disease (NAFLD) and end stage liver disease (ESLD). Indeed, sarcopenia is reported to be present in approximately 25–70% of ESLD patients (Kim et al., 2017) and negatively impacts quality of life, significantly increases mortality risk, and may adversely affect the outcome of liver transplantation (Carey et al., 2017). Despite these implications, the underlying mechanisms of sarcopenia within CLD remain relatively unknown.

To truly understand sarcopenia, it is imperative to understand the mechanistic drivers that underpin these changes in skeletal muscle. As such, a wealth of research outside the liver field has been based on percutaneous muscle biopsies to obtain muscle tissue and investigate the morphological, molecular, and genetic mechanisms of primary sarcopenia (Patel et al., 2015). While these studies often included older individuals (>65 years), the participants are typically “healthy” with minimal co-morbidities. As such, the muscle biopsy procedure in these non-clinical settings poses minimal risk and few complications. However, in clinically vulnerable cohorts there are many additional considerations and risks which must be mitigated, and, in many cases, there are contraindications for muscle biopsies (Wilson et al., 2017). Despite this, when a careful and considered approach is taken, percutaneous muscle biopsies can be completed in clinically challenging populations such as patients with cancer (MacDonald et al., 2015; Aversa et al., 2016) and those suffering from chronic inflammatory diseases (Dalakas, 2002; Van Langenberg et al., 2014; Boutrup et al., 2018). However, very few studies have investigated the molecular mechanisms of sarcopenia within patients with CLD using muscle biopsies (Tsien et al., 2015) and much of previous work is limited to cell and animal based models (Dasarathy et al., 2004, 2007). Thus, our understanding of the drivers of sarcopenia within CLD remains very limited. It is likely that the biological basis of these conditions and the consequent significant safety concerns has evoked caution.

Liver failure and coagulopathy present in ESLD and complications of CLD such as portal hypertension alter the usual clotting pathways, resulting in thrombocytopaenia and coagulopathy (Ginès et al., 2021). This results in an increased predisposition to bleeding and poor clotting. Other complications include hepatic encephalopathy (HE) which can affect cognition and the capacity to consent (Pantham and Mullen, 2017). In addition, as the liver is essential for glucose homeostasis, liver dysfunction can alter glucose uptake in both liver and skeletal muscle, increasing glycogenolysis and insulin resistance (Petrides and DeFronzo, 1989). CLD can often be accompanied by other co-morbidities, such as Diabetes Mellitus (Jepsen, 2014). Thus, these patients are often on a number of treatments, including insulin and oral hypoglycaemic medications which, if not correctly mitigated, pose significant complications for muscle biopsies. Specifically, prolonged fasting without dosage alterations or omissions can result in symptomatic hypoglycaemia, adverse events and consequently halting of the procedure (Hochberg et al., 2019). The stress response may further compound hypoglycaemia by decreasing serum glucose concentrations (Gibbons et al., 2012). In addition to medication concerns, within CLD, the presence of NAFLD is often associated with higher amounts of subcutaneous fat (Nishikawa et al., 2021), which can add an additional challenge to the subcutaneous muscle biopsy procedure. Therefore, the purpose of this study was to establish a safe and effective protocol for percutaneous muscle biopsies in patients with CLD, to instigate the necessary invasive mechanistic research.

## Materials and Methods

### Patient Details

A total of 47 patients with ESLD and 9 patients with non-cirrhotic NAFLD. Patients were recruited from the Liver Unit, Queen Elizabeth Hospital (Birmingham, United Kingdom), as part of a larger, prospective observational study (ClinicalTrials.gov Identifier: NCT04734496). Within this study, multiple visits occurred, two of which included the possibility of a muscle biopsy (separated by 6 months). Full patient demographics alongside comorbidities and CLD related complications can be seen in Table 1. Primary aetiology and disease severity [as defined by Model for End Stage Liver Disease (MELD) score and Child-Pugh scoring] can be seen in Table 2. The study was approved by the Health Research Authority – West Midlands Solihull Research Ethics Service Committee Authority (REC reference: 18/WM/0167). All patients provided written informed consent.

**TABLE 1:** Patient characteristics, co-morbidities, and CLD related complications for both ESLD and non-cirrhotic NAFLD groups, as well as combined.

Mean (±SD)	ESLD	Compensated NAFLD	Combined
*N*	47	9	56
Age (years)	54.7 (10.4)	61.3 (7.9)	55.8 (10.2)
Sex (m/f)	31/16	6/3	31/16
Height (cm)	172.0 (11.9)	168.3 (8.4)	171.4 (11.5)
Weight (kg)	89.0 (21.4)	107.3 (33.2)	92.1 (24.3)
BMI (kg/m^2^)	29.5 (6.5)	34.5 (8.0)	30.3 (6.9)
Body fat%	29.2 (10.7)	38.1 (13.3)	30.7 (11.5)

***N* (%)**	**ESLD**	**Compensated NAFLD**	**Combined**

**Co-morbidities**			
Diabetes Mellitus (DM)	12 (25)	4 (44)	16 (28)
DM (insulin dependent)	9 (19)	1 (11)	10 (18)
Chronic kidney disease	8 (17)	0	8 (14)
Chronic obstructive pulmonary disease	8 (17)	1 (11)	9 (14)
Cardiovascular disease	4 (8)	0	4 (7)
Hypertension	14 (30)	4 (44)	18 (32)
**CLD related complications**			
Hepatic encephalopathy	27 (56)	0 (0)	27 (48)
Portal hypertension	43 (91)	0 (0)	43 (76)

*Characteristic data shown as mean average (±SD). Co-morbidity and CLD related complications data as N (% of respective group).*

**TABLE 2:** Disease aetiology for the liver disease group.

Liver disease aetiology	*N* (%)	MELD (±SD)	Child-Pugh score (±SD)
Alcohol related liver disease	25 (45)	14.6 (4.9)	8.7 (1.1)
Cirrhotic NAFLD	6 (11)	12.4 (5.6)	7.0 (1.5)
Primary sclerosing cholangitis	9 (16)	11.6 (2.5)	6.3 (1.5)
Primary biliary cirrhosis	5 (9)	11.5 (1.7)	6.7 (0.9)
Other causes	2 (3)	10.5 (1.0)	6.0 (0.7)
Compensated NAFLD	9 (16)	N/A	N/A
Combined	56 (100)	13.2 (4.4)	7.7 (1.6)

*Data shown as N (% of cohort) and as mean average (±SD) for MELD and Child-Pugh score.*

### Muscle Biopsy Protocol

Percutaneous muscle biopsies were obtained *via* the modified Bergström technique from mid vastus lateralis (VL) in fasted patients *via* full aseptic technique (Bergstrom, 1975). The biopsy site was obtained as 50% of the distance between the greater trochanter and mid patellar, and 50% between the medial and lateral borders of the VL (obtained *via* ultrasonography). The VL offers a safe option for muscle biopsies due to limited motor units and the lack of major blood vessels or nerves traversing the biopsy site (Andrew Shanely et al., 2014). In brief, the skin was cleaned with povidone-iodine antiseptic and approximately 5–10 ml of 1% lidocaine was utilised to fully anesthetise the biopsy site. A 5–8 mm incision was made through the skin, and any initial bleeding was stemmed by applying pressure with sterile gauze. A deeper incision was then made through the muscle fascia and into the muscle tissue, allowing a path for a 5 mm Bergström needle. Suction was applied to the Bergström needle *via* catheter and 100 ml syringe. Typically, three passes were attempted to obtain tissue, with a maximum of four passes. Operators would aim to obtain tissue within 15–30 s during each pass. Once completed, the wound was sealed, dressed and a cold compress was applied. Patients were supplied with a post biopsy aftercare leaflet and additional dressings were provided.

**FIGURE 1:**
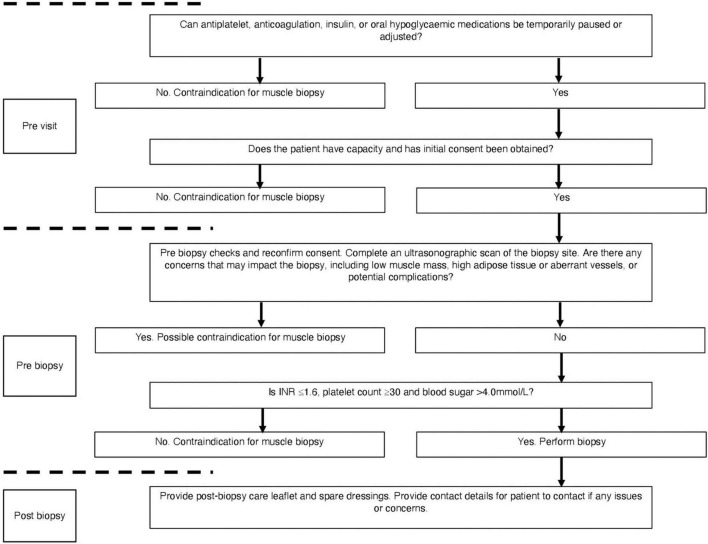
Flowchart for safety considerations prior to muscle biopsy. Checks occur at pre-visit, pre-biopsy, and post-biopsy follow ups.

To ensure the safety of the patient and maximise the success of the biopsy procedure, we developed a detailed protocol which considered risk factors and established possible mitigation (Figure 1). Two weeks prior to their muscle biopsy visits, a medical doctor checked all medications including any antiplatelet, anticoagulation, insulin, or oral hypoglycaemic medications. We applied a strict ruling whereby no biopsy procedure would occur if pausing of any medication could incur significant risk (e.g., warfarin for venous thromboembolism). However, when feasible, medication was paused for a minimum of 3 days in advance of the biopsy visit, in accordance with drug half-life and national guidelines for gastroenterology endoscopic procedure (Veitch et al., 2016). In addition to anticoagulation and antiplatelet medication, a medical doctor also screened for insulin use and contacted patients in advance to advise on adjustment of their insulin doses the day prior to and day of the biopsy. This followed national guidelines for GI endoscopic procedure and radiology procedural guidelines (Runyon, 2009; Kurup et al., 2015; Veitch et al., 2016). For those with HE, only those who were deemed to have capacity to consent for the procedure were included. To ensure capacity and that the patient understood the procedure, consent was obtained on multiple occasions (prior to the visit, on arrival, and pre biopsy).

**FIGURE 2:**
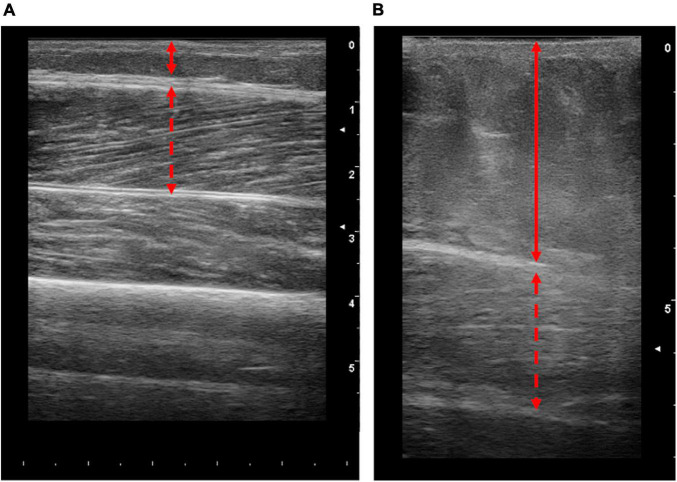
Representative longitudinal ultrasound images of the vastus lateralis (VL) from patients with end stage liver disease. Images obtained from patients with lower **(A)** and higher **(B)** body fat percentages (10.5 and 46%, respectively). Solid lines represent the thickness of adipose tissue and dashed red arrow represents the thickness of the VL. Scale on the right-hand side of each image represents cm.

On the biopsy visit, a stringent safety checklist was utilised to ensure that every patient was adequately risk assessed. We devised a Local Safety Standards for Invasive Procedures (LocSSIP) checklist [similar to a World Health organization (WHO) checklist used in surgery] for performing the muscle biopsy procedure. The LocSSIP reconfirms capacity, consent, notes any allergies and medications, as well as confirming any medications that have been temporally paused for the procedure. At this stage, a pre-biopsy check of INR and platelets occurred, whereby a cut-off of INR ≥ 1.6 (normal value < 1.2) and platelet count ≤30 × 10^9^/L (normal range 50–450 × 10^9^/L) would contraindicate biopsy; in concordance with national and local radiology guidance for procedures entailing a similar risk profile (Runyon, 2009; Kurup et al., 2015). If all safety concerns were satisfied, a point of care blood sugar (BM) check occurred, as those with diabetes and CLD are at an increased risk of hypoglycaemia due to fasting and liver dysfunction (Ginès et al., 2021). The cut-off point of <4.0 mmol/L was set as contraindication for muscle biopsy. Finally, as part of the wider study, ultrasonographic assessment of the VL occurred during each visit and as such helped identify the optimal muscle biopsy site. The captured images were also interpreted for VL size and the degree of subcutaneous adipose tissue and hence the depth from skin to muscle tissue (Figure 2). The scans also enabled assessment for any aberrant vessels, or potential complications. Although the procedure itself was “blind,” the biopsy site was visually assessed for morphology and any possible contraindications. In addition to those mentioned above, other contraindications at the pre-biopsy check stage (Figure 1) would include any pre-existing swelling, infection or injury surrounding the proposed biopsy site or lower limb, systemic acute illness or haemodynamic instability. The protocol was to immediately stop the procedure if concerns were raised (from either the operator or patient).

## Results

### Feasibility of Muscle Biopsies

**FIGURE 3:**
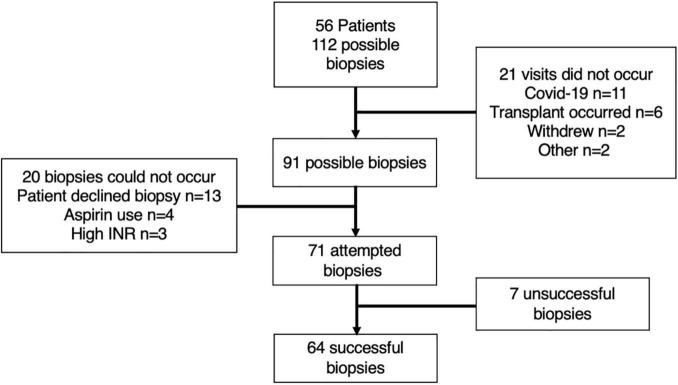
Schematic representation of included patients and visits. The reasons for either the non-occurrence of visits, contraindications for biopsy and unsuccessful muscle biopsies are explained within the attached boxes.

As 56 patients were recruited, a maximum of 112 scheduled muscle biopsy visits could have occurred (i.e., 2 possible visits for each patient) (Figure 3). A total of 21 of these visits did not occur, primarily due to cancellations associated with COVID-19 or the patient undergoing recent liver transplantation, thus leaving 91 possible visits (Figure 3). In the visits that did occur, 85% of patients agreed to undergo a muscle biopsy (48/56), with only 13 biopsies not occurring due to patients declining. Including all visits whereby the patient agreed and provided consent, only 9% of biopsies could not occur due to medical considerations, including high INR and the use of aspirin (Figure 3).

### Efficacy of Muscle Biopsies

A total of 71 muscle biopsies were consented and deemed safe to attempt; 64 of which were successful (i.e., yielded tissue) (90% of attempted) (Figure 3). The average yield of muscle tissue from all attempted biopsies (*n* = 71) was 89.5 ± 57.7 mg. However, if only successful biopsies are considered (*n* = 64), then the yield was 98.1 ± 52.9 mg (18–226 mg).

### Haematology

The mean average platelet count for all patients (i.e., all patients included within the study, not just those consented to biopsy) was 126 ± 71 (range 27–380) and mean average INR was 1.3 ± 0.4 (range 0.9–3.2). Only one patient would have been below the platelet cut-off (<30), whilst six patients were above the INR cut-off (>1.6) for muscle biopsy. Whereas, if only those who consented to biopsy were considered, then no patient was below the platelet cut-off, and only three had high INR (Figure 3).

### Adverse Events

A total of 71 muscle biopsies were performed, and we report only a single complication associated with the biopsy. Although the individual had an INR and platelet count within the acceptable range, a superficial haematoma at the site of biopsy occurred. The procedure itself was uneventful. The issue self-resolved within a week and did not require any medical management, or hospital admission. There were no reports of localised infection, or any other muscle biopsy associated complication post biopsy.

## Discussion

The data herein show that percutaneous biopsies of the VL are feasible, safe, and yielded muscle tissue in patients with CLD. Our safety protocol provided a stringent approach to the procedure which mitigated against, and significantly reduced the possible risk of adverse events. Thus, we believe that this study provides a platform to support future research into the mechanistic drivers of sarcopenia within CLD.

With regard to the feasibility of muscle biopsies, we showed that 85% of patients (48/56) agreed and consented to the procedure. As such, this meant that only 13 biopsies were missed due to lack of consent. It is worth noting that of the 8 patients who declined, 5 of these patients refused the biopsy at both visits (i.e., accounts for 10 of the 13 missed biopsies). Many investigators will attest to the challenge of recruitment for a voluntary invasive procedure, such that there will always be those who decline. Nonetheless, our data demonstrate that the concept of a muscle biopsy appears to be not overly daunting for those with CLD. It is possible that this patient group may have become accustomed to invasive procedures due to their diagnosis and treatment (i.e., liver biopsy, ascites drains, and regular blood tests). Importantly, within the consenting patients, only 7 (9%) were deemed unsafe to complete the procedure; due to coagulopathy, or in those unable to alter their anticoagulation/antiplatelet medication. Thus, even when stringent cut offs are utilised, the large majority of patients are eligible for the muscle biopsy procedure. Another potential concern for feasibility was the impact of HE on the patient’s ability to consent. To navigate this issue, we ensured that consent was obtained on multiple occasions (i.e., prior to the visit, on arrival, and pre-biopsy). Nonetheless, despite ∼50% of patients having some degree of HE, we had no concerns due to lack of capacity to consent to the biopsy based on the clinical judgement of the research team.

A total of 71 muscle biopsies were consented to and deemed safe. A total of 90% of these attempts were successful (i.e., yielded muscle tissue). Unfortunately, seven attempted muscle biopsies were unsuccessful. It is worth noting that during this research study, junior colleagues were completing training on the muscle biopsy procedure and 3/7 of the unsuccessful procedures occurred due to this reason. One unsuccessful attempt was due to the patient feeling unwell and anxious following the anaesthetic, i.e., before an incision was made, so the procedure was aborted. Finally, the remaining three unsuccessful attempts included patients with high amounts of subcutaneous adipose tissue (mean body fat 41.2%), highlighting the procedural challenges associated with these populations. Indeed, as the procedure utilised a Bergström needle, there are practical limitations of this method, i.e., the limited needle length. Thus, whilst the needle itself may reach the muscle tissue; the procedure requires the needle window to be sufficiently within the muscle to obtain a sample. Therefore, in patients where subcutaneous adipose tissue is high, i.e., ∼5 cm adipose thickness (Figure 2B), the Bergström approach may not be suitable. As such, while ultrasound was not intentionally a component of the protocol, we suggest that the ultrasonographic assessment prior to biopsy is critical. Nonetheless, 64 muscle biopsies were successful with an average yield of 98.1 ± 52.9 mg (range of 18–226 mg). Molecular and RNA analysis of muscle tissue typically utilises ∼20 mg (Franchi et al., 2014) and ∼5–10 mg (Din et al., 2019) of wet weight tissue, respectively. Thus, all but one biopsy would be appropriate for western blot analysis, and all samples would be appropriate for RNA analysis. Collectively, this demonstrates that percutaneous muscle biopsies can be successful in patients with CLD.

Muscle biopsies have long been utilised for the molecular investigation of muscle tissue within a range of ages and conditions (Goodpaster et al., 2000; Tarnopolsky et al., 2011; Barthelemy et al., 2020). However, within any population there can be challenges associated with the muscle biopsy procedure. For example, in obese patients where adipose tissue is high (Tarnopolsky et al., 2011; Wang et al., 2011), or in individuals with low muscle mass such as both young and elderly patients (Ekblom, 2017; Wilson et al., 2018). The procedure can be further complicated when considering medication such as insulin and oral hypoglycaemia medications such as those with type 2 diabetes (Wang et al., 2011). Likewise, our study demonstrates that there are several safety considerations to mitigate in patients with CLD or NAFLD, specifically coagulopathy. There has been a reluctancy to perform muscle biopsies within this high-risk group. Surprisingly, we reported only one adverse event (haematoma), which is in line with previous research (Tarnopolsky et al., 2011). This highlights that appropriate patient selection, stringent pre biopsy preparation and good technique mitigates against potential risk. We also report no incidence of localised infection associated with the biopsy procedure. It is likely that the clear post procedure instructions were important to reduce the risk of complications and infection. In addition, patients had regular clinic visits as well as follow-up research visits; thus, if any issues arose, these could be reported and appropriately treated. For future research, we would recommend that a follow-up telephone call should be made in the case where follow-up visits do not occur. Finally, although we applied this safety protocol in specific ESLD and NAFLD groups, it is likely that the same safety protocol could be applied to other liver disease scenarios. Nonetheless, despite our success, to further improve the procedure a true ultrasound guided approach could be utilised to provide visual feedback during the biopsy.

## Conclusion

We demonstrate that percutaneous muscle biopsies in patients with CLD are feasible and can yield sufficient muscle tissue. Indeed, only 15% of biopsies were declined by the patient, and 90% of the 71 attempted biopsies were successful with only one reported adverse event. It is likely that the success stems from the careful and considered approach to muscle biopsies in this population, considering medications, comorbidities, and mitigating against potential complications. As such, it provides grounds for further mechanistic work into the molecular drivers behind sarcopenia in CLD.

## Data Availability Statement

The raw data supporting the conclusions of this article will be made available by the authors, without undue reservation.

## Ethics Statement

The studies involving human participants were reviewed and approved by the Health Research Authority – West Midlands Solihull Research Ethics Service Committee Authority (REC reference: 18/WM/0167). The patients/participants provided their written informed consent to participate in this study.

## Author Contributions

JQ, AD, FW, and SA acquired the data. JQ and AD completed further analysis of the data contained within the manuscript. JQ and AD jointly constructed initial and final drafts. JQ, AD, FW, LB, CG, JL, MA, and AE aided in the production of the protocol discussed within the manuscript. All authors edited and approved the final manuscript.

## Author Disclaimer

The views expressed are those of the author(s) and not necessarily those of the NHS, the NIHR or the Department of Health and Social Care.

## Conflict of Interest

The authors declare that the research was conducted in the absence of any commercial or financial relationships that could be construed as a potential conflict of interest. The reviewer BS declared a past collaboration with one of the authors, LB, to the handling editor.

## Publisher’s Note

All claims expressed in this article are solely those of the authors and do not necessarily represent those of their affiliated organizations, or those of the publisher, the editors and the reviewers. Any product that may be evaluated in this article, or claim that may be made by its manufacturer, is not guaranteed or endorsed by the publisher.
